# Complementary Role of P2 and Adenosine Receptors in ATP Induced-Anti-Apoptotic Effects Against Hypoxic Injury of HUVECs

**DOI:** 10.3390/ijms20061446

**Published:** 2019-03-22

**Authors:** Catherine Feliu, Hélène Peyret, Gael Poitevin, Yoann Cazaubon, Floriane Oszust, Philippe Nguyen, Hervé Millart, Zoubir Djerada

**Affiliations:** 1 Department of Pharmacology, E.A.3801, SFR CAP-santé, Reims University Hospital, 51, rue Cognacq-Jay, 51095 Reims CEDEX, France; catherine.feliu@univ-reims.fr (C.F.); helene.peyret@univ-reims.fr (H.P.); yoann.cazaubon@univ-reims.fr (Y.C.); floriane.oszust@univ-reims.fr (F.O.); herve.millart@univ-reims.fr (H.M.); 2 Laboratory of Hematology, E.A.3801, SFR CAP-santé, Reims University Hospital, 51, rue Cognacq-Jay, 51095 Reims CEDEX, France; gael.poitevin@univ-reims.fr (G.P.); philippe.nguyen@univ-reims.fr (P.N.)

**Keywords:** endothelial cells, hypoxic stress, purinergic signaling, ATP, adenosine

## Abstract

Background: Vascular endothelial injury during ischemia generates apoptotic cell death and precedes apoptosis of underlying tissues. We aimed at studying the role of extracellular adenosine triphosphate (ATP) on endothelial cells protection against hypoxia injury. Methods: In a hypoxic model on endothelial cells, we quantified the extracellular concentration of ATP and adenosine. The expression of mRNA (ectonucleotidases, adenosine, and P2 receptors) was measured. Apoptosis was evaluated by the expression of cleaved caspase 3. The involvement of P2 and adenosine receptors and signaling pathways was investigated using selective inhibitors. Results: Hypoxic stress induced a significant increase in extracellular ATP and adenosine. After a 2-h hypoxic injury, an increase of cleaved caspase 3 was observed. ATP anti-apoptotic effect was prevented by suramin, pyridoxalphosphate-6-azophenyl-2′,4′-disulfonic acid (PPADS), and CGS15943, as well as by selective A2A, A2B, and A3 receptor antagonists. P2 receptor-mediated anti-apoptotic effect of ATP involved phosphoinositide 3-kinase (PI3K), extracellular signal-regulated kinases (ERK1/2), mitoK_ATP_, and nitric oxide synthase (NOS) pathways whereas adenosine receptor-mediated anti-apoptotic effect involved ERK1/2, protein kinase A (PKA), and NOS. Conclusions: These results suggest a complementary role of P2 and adenosine receptors in ATP-induced protective effects against hypoxia injury of endothelial. This could be considered therapeutic targets to limit the development of ischemic injury of organs such as heart, brain, and kidney.

## 1. Introduction

Vascular endothelium is a cell monolayer at the interface of blood flow and underlying tissues and organs [1,2]. Vascular endothelial injury, related to ischemia, generates oxidative stress and apoptotic cell death, which induce a dysfunction and favor the development of atherosclerosis [1,2,3,4]. A study describing the time course of apoptosis in different cell types in the heart during ischemia/reperfusion demonstrated that endothelial cells were the first to be affected [5]. Endothelial apoptosis plays a major role in the progression of ischemic injury and radial spread of apoptosis to surrounding cardiomyocytes [4,5]. In another context, endothelial protection has been shown to be essential in maintaining normal cardiac function after transplantation, mainly by controlling coronary circulation [6]. Parolari et al. [6] have shown that both myocardial and endothelial protections are necessary to progress from myocardial to cardiac protection. Endothelial injury is presented as the initiator of deleterious cascades of organ damage [7]. Pharmacological protection against vascular endothelial apoptosis could limit the development of ischemic injury of various organs including heart, brain, and kidney. 

Many endogenous signaling molecules have been described to induce protection against cardiac stress (hypoxic or ischemic stress). Among them, extracellular nucleotides such as adenosine tri-phosphate (ATP) and nucleosides, such as adenosines, are secreted by endothelial cells and cardiomyocytes during ischemia [8]. Purinergic signaling plays an important role in the cardiovascular system [8,9]. It regulates vascular tone as well as growth of vascular smooth muscle cells and endothelial cells. Purinergic system is involved in vascular remodeling, coagulation, and inflammation [8,10]. It is also implicated in cardiac inotropy and cardioprotection [8,11]. Recent studies have established the involvement of P1/P2 receptors in cardioprotection during ischemia [8,12,13,14]. 

The nucleotides/nucleosides secreted at high concentration in the interstitium during cardiac ischemia have been reported to be adenosine 5′-triphosphate (ATP) and adenosine [12,13,15]. Thus, the purpose of this study was to evaluate the role of ATP and adenosine receptors in endothelial cells during hypoxia-reoxygenation. For this, we confirmed nucleotides/nucleosides release during hypoxia-reoxygenation by an appropriate method able to simultaneously quantify the different targeted compounds in extracellular media. Then, we have studied the induction of cellular protection induced by extracellular signaling of ATP and adenosine as natural agonists. The use of natural agonists is necessary to approach in vivo physio-pathological conditions such as the metabolism and cellular turn-over of nucleotides.

## 2. Results

### 2.1. Hypoxic Stress Induces both ATP and Adenosine Release from Endothelial Cells

**Figure 1:**
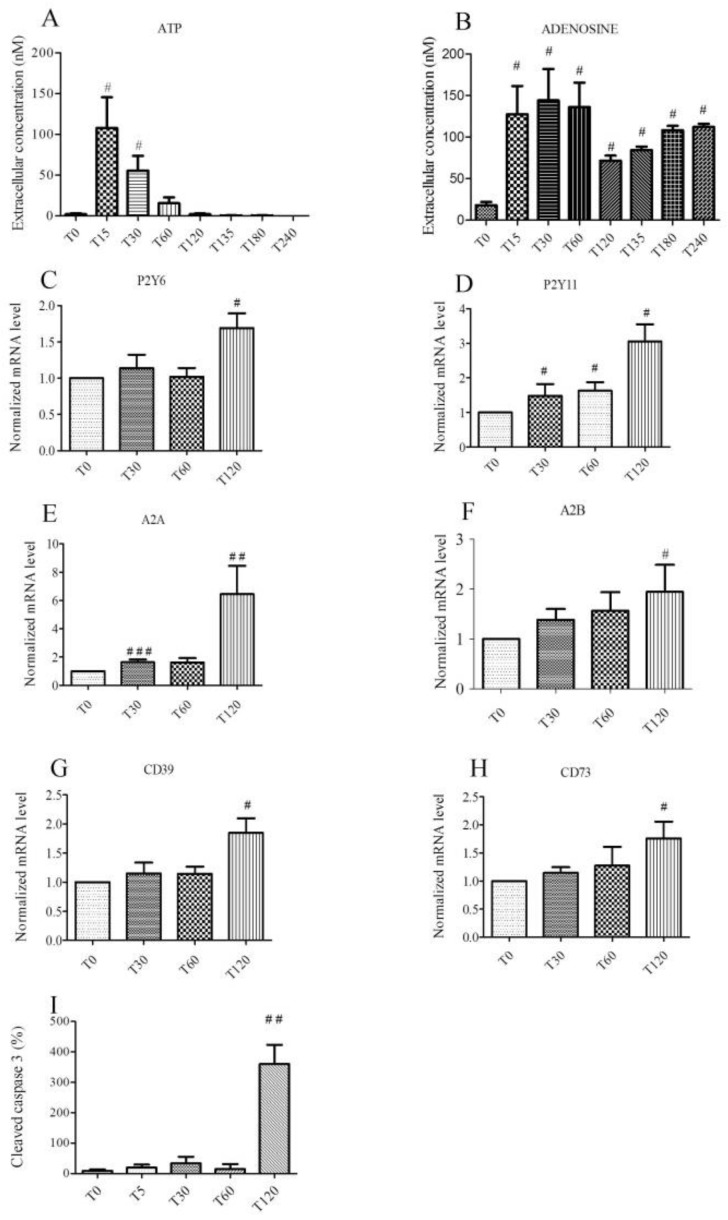
Effect of a 2-h hypoxia on endothelial cells. Extracellular ATP (**A**) and adenosine (**B**) concentrations are expressed in nM, during hypoxia (T0–T120) and at re-oxygenation (T135–T240). Overexpression of mRNA for P2Y6 (**C**) and P2Y11 (**D**) receptors, adenosine receptors A2A (**E**), A2B (**F**), and ectonucleotidases CD39 (**G**) and CD73 (**H**) expressed with normalized mRNA levels using the following formula: 2^−ΔΔCT^ as a function of time. Relative expression of cleaved caspase 3 by immunoblotting during hypoxia (**I**). Results are expressed as means ± sem (*n* = 6/group). #: *p <* 0.05, ##: *p <* 0.01, ###: *p <* 0.001 compared to T0 group.

The release of adenosine, adenosine 5′-monophosphate (AMP), uridine 5′-monophosphate (UMP), Adenosine 5′-diphosphate (ADP), Uridine 5′-diphosphate (UDP), Adenosine 5′-triphosphate (ATP), and Uridine 5′-triphosphate (UTP) from human umbilical vein endothelial cells (HUVECs) into the extracellular medium (in 500 µL of supernatant) during hypoxia. Re-oxygenation has been studied at different times (T0, T15, T30, T60, T120, T135, T180, and T240 min) using liquid chromatography coupled with high resolution mass spectrometer (LC-HRMS). There was a significant and concomitant increase in ATP, ADP, AMP, and adenosine concentrations during hypoxia. Contrary to the nucleotides, adenosine concentrations remained steady during re-oxygenation (Figure 1, Supplementary file 1). Concentrations of ATP and adenosine were the most important during the experiment (Figure 1A,B). Extracellular concentration of ATP increased significantly for 15 min (108.4 ± 37.4 nM, *p* < 0.05) after the onset of hypoxia compared to the control group (T0: 2.0 ± 1.0 nM). Concentration of ATP decreased rapidly after 30 min (55.2 ± 17.7 nM, *p* < 0.05 vs. control group T0, and T60: 15.8 ± 5.9 nM, *p* < 0.05) and was undetectable after 60 min of hypoxia. Extracellular concentration of adenosine increased significantly (*p* < 0.05) during hypoxia and re-oxygenation (T15: 127.2 ± 33.6 nM, T30: 142.2 ± 37.4 nM, T60: 134.7 ±26.1 nM vs control group T0: 18.7 ± 3.7 nM). After 60 min, extracellular concentration of adenosine decreased but remained significantly (*p* < 0.05) higher than the concentration of the control group (T120: 71.1 ±7.5 nM). During the subsequent re-oxygenation period, adenosine concentration remained stable (T135: 82.3 ±3.7 nM, T180: 108.5 ±3.7 nM, T240: 112.3 ±3.7 nM), while ATP was undetectable.

### 2.2. Hypoxic Stress Induces Overexpression of P2Y_6_, P2Y_11_, A_2A_, A_2B_, CD 39, and CD 73 mRNAs

In our model, the expression of P2Y receptors, adenosine receptors, and ectonucleotidases mRNA was studied during two hours of hypoxia. P2Y_1_, P2Y_2_, P2Y_4_, P2Y_6_, P2Y_11_, P2X_7_, P2X_4_, A_2A_, A_2B_, and A_3_ receptors were expressed in HUVECs (data not shown). The relative expression to the control group (T0) was estimated as normalized mRNA levels using the following formula: 2^−ΔΔC^_T_ of different conditions. Increased expression of mRNA was significant (*p* < 0.05) for P2Y_6_ (T120: 1.69 ± 0.20), P2Y_11_ (T30: 1.48 ± 0.34, T60: 1.630 ± 0.24 and T120: 3.05 ± 0.49), A_2A_ (T30: 1.63 ± 0.19 and T120: 6.44 ± 1.99), and A_2B_ (T120: 1.943 ±0.54). Increased expression of mRNA was also significant (*p* < 0.05) for ectonucleotidases CD39 T120: 1.849 ± 0.247 and CD73 T120: 1.761 ± 0.297, involved in the conversion of ATP into adenosine (Figure 1C–H). 

### 2.3. Hypoxia Promotes Apoptosis in Endothelial Cells

HUVECs were exposed to simulated hypoxia for two hours. Cells were harvested to analyze the expression of cleaved caspase 3 at different times. Stress related to hypoxia resulted in a significant increase in apoptosis of HUVECs. Relative expression to the control group (T0) of cleaved caspase 3 was increased after 2 h of hypoxia: T120: 1029 ± 181.3% vs control group T0 100 %, *n* = 6, *p* < 0.05, Figure 1I). The lactate dehydrogenase activity (LDH) measured after 2 h of hypoxia experiment did not change significantly (Supplementary file 2). Hypoxia induced apoptosis without inducing LDH release.

### 2.4. Extracellular ATP and Adenosine Induced an Anti-Apoptotic Effect

**Figure 2:**
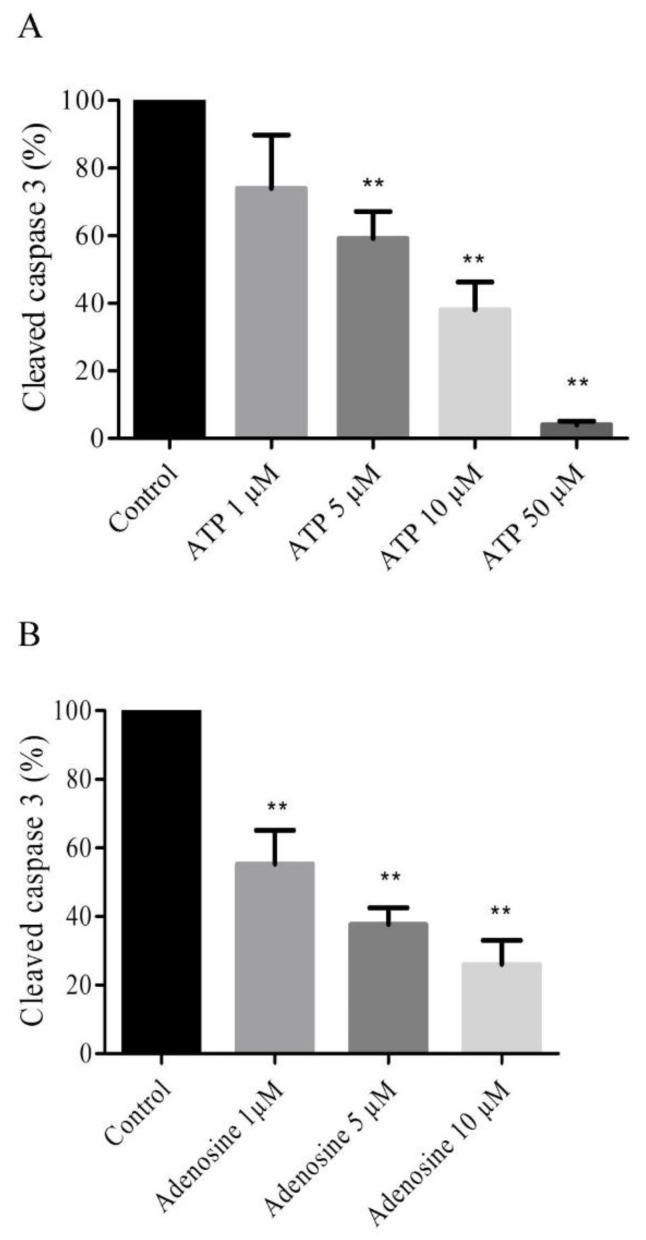
ATP and adenosine induced an anti-apoptotic effect. Cells were treated with ATP 1 µM, 5 µM, 10 µM, and 50 µM (**A**) and adenosine 1 µM, 5 µM, and 10 µM (**B**). Results are expressed as means ± sem (*n* = 6/group) of relative caspase 3 expression (%) in the HUVECs after 2 h of hypoxia. ** *p* < 0.01 compared to control without any treatment.

An anti-apoptotic effect of extracellular ATP was assessed in HUVECs treated with ATP (1 µM, 5 µM, 10 µM and 50 µM) for two hours of hypoxia. A significant decrease (*n* = 6, *p* < 0.05) in the relative expression of cleaved caspase 3 was observed in cells treated with 5 µM ATP (59 ± 7.9%), 10 µM (38 ± 8.2%) and 50 µM (4 ± 1.1%) versus control group 100% (Figure 2A). The anti-apoptotic effect of extracellular adenosine was also assessed in HUVECs treated with adenosine (1 µM, 5 µM and 10 µM) before 2 h of hypoxia. A significant decrease (*n* = 6, *p* < 0.05) of relative expression of cleaved caspase 3 was observed in cells treated with 1 µM adenosine (55.33 ± 9.82%), 5 µM (37.67 ± 4.91%), and 10 µM (26 ± 7%) vs. a control group of 100% (Figure 2B).

### 2.5. Protective Effects of Extracellular ATP Are Mediated by P2 and Adenosine Receptors

**Figure 3:**
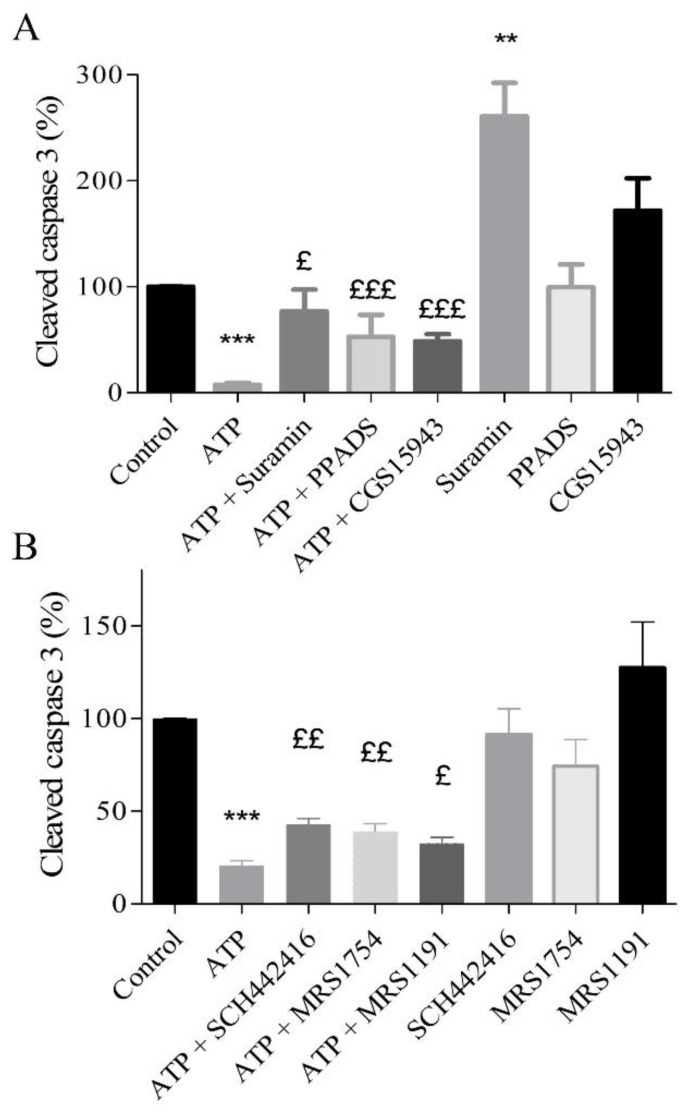
Anti-apoptotic effect of extracellular ATP mediated by both P2 and adenosine receptors (**A**). HUVECs, exposed to a simulated hypoxia during 2 h, were treated with ATP 10 µM, P2 antagonist (10 µM suramin and PPADS), and adenosine antagonist (1 µM CGS15943). Subtypes of adenosine receptors were studied using selective receptors antagonists (**B**) of A2A (10 µM SCH442416), A2B (0.1 µM MRS1754), and A3 (10 µM MRS1191) receptors. Data are means ± sem (*n* = 6/group) of relative caspase 3 expression (%) in HUVECs after 2 h of hypoxia. ^£^ *p* < 0.05, ^££^ *p* < 0.01, ^£££^ *p* < 0.001 compared to 10 µM ATP group. ** *p* < 0.01, *** *p* < 0.001 compared to control without treatment.

The role of P2 and P1 receptors involved in the anti-apoptotic effect of extracellular ATP was investigated using a panel of selective antagonists (Table 1). Treatment was performed with 10 µM ATP and P2 antagonists, 10 µM suramin or pyridoxalphosphate-6-azophenyl-2’,4’-disulfonic acid (PPADS) or adenosine antagonist (1 µM CGS15943) (Figure 3A). The anti-apoptotic effect of 10 µM ATP was partially abolished by 10 µM suramin (76.75 ± 20.70%), by 10 µM PPADS (52.8 ± 20.6%), or by CGS15943 (48.61 ± 6.87%) compared to cells treated with 10 µM ATP alone (7.5 ± 2.1%; *n* = 9, *p* < 0.05). These results suggest that P2 and adenosine receptors are involved in the anti-apoptotic effect triggered by ATP (Figure 3A). Compared to control, only suramin increased cleaved caspase 3 (*p* < 0.01).

### 2.6. P2 Receptor-Mediated Anti-Apoptotic Effect of ATP Involves PI3K, MEK/ERK1/2, mitoK_ATP_, and NOS Pathways

To assess the signaling pathways involved in the P2 receptor-mediated effect, HUVECs were treated with 1 µM CGS15943 and different inhibitors (Table 1): 10 µM U0126 (MEK/ERK1/2), 10 µM LY294002 (PI3K), 100 µM 5-hydroxydecanoate (5-HD, mitoK_ATP_ channel), and 10 µM N(ω)-nitro-L-arginine methyl ester (L-NAME, NOS), 20 µM H89 (PKA), and 5 µM indomethacin (Cyclooxygenase COX). The anti-apoptotic effect of 10 µM ATP and 10 µM ATP + 1 µM CGS15943 was unaffected by blocking PKA and COX pathway (data not shown). Compared to the control, we observed no difference with U0126, H89, L-NAME, or 5-HD, while no significant increase was observed with LY294002 (Supplementary file 3).

**Figure 4:**
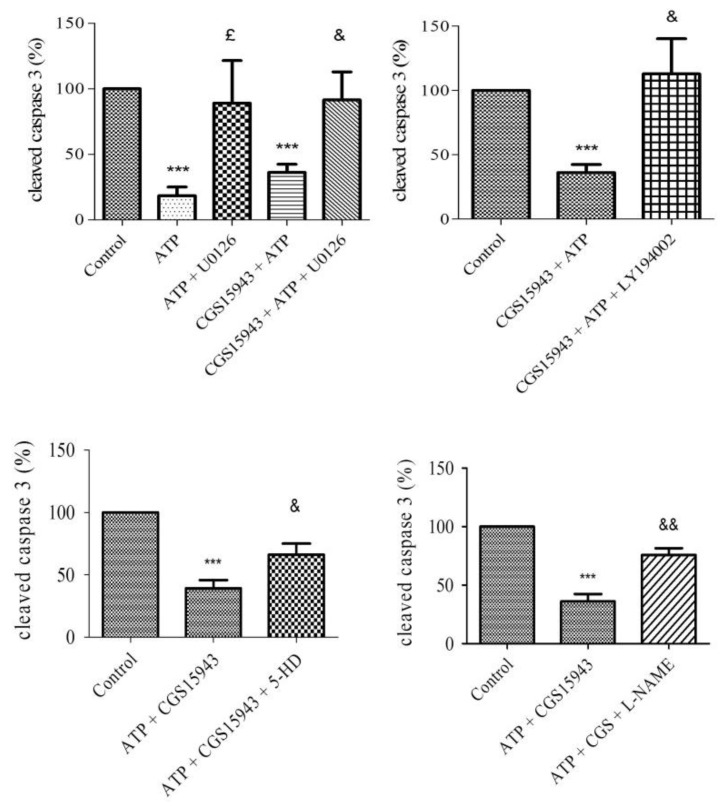
Signaling pathways involved in the P2-mediated anti-apoptotic effect of ATP. HUVECs were treated with different antagonists: 10 µM U0126 (MEK/ERK1/2), 10 µM LY294002 (PI3K), 100 µM 5-HD (mitoK_ATP_), and 10 µM L-NAME (NOS). Then, cells were treated with 10 µM ATP and 1µM CGS15943 and submit to 2 h of hypoxia. Data are means ± sem (*n* = 6/group) of relative caspase 3 expression (%) in HUVECs after 2 h of hypoxia. ^£^ *p* < 0.05, compared to 10 µM ATP group. ^&^ *p* < 0.05, ^&&^ *p* < 0.01 compared to 10 µM ATP + 1 µM CGS15943 group. *** *p* < 0.001 compared to the control.

The anti-apoptotic effect (relative to control) of 10 µM ATP (18 ± 6.6%) during hypoxia was abolished by blocking MEK/ERK1/2 pathway (89 ± 32.5%, *p* < 0.05). This result was also observed in cells co-treated with ATP and CGS15943 vs. ATP + CGS15943 + U0126. The anti-apoptotic effect of 10 µM ATP and 1 µM CGS15943 (39.23 ± 6.52%) was significantly and partially abolished by blocking MEK/ERK1/2 (91.57 ± 21.30%), PI3K (112.8 ± 27.42%), mitoK_ATP_ (66.17 ± 8.88%), and NOS (75.80 ± 5.7%) pathway (*n* = 6, *p* < 0.05), with 10 µM U0126 (MEK/ERK1/2), 10 µM LY294002 (PI3K), 100 µM 5-HD (mitoK_ATP_), and 10 µM L-NAME (NOS), respectively (Figure 4). This suggests the involvement of MEK/ERK1/2, PI3K, mitoK_ATP_ channel, and NOS in P2 signaling.

### 2.7. A_2A_, A_2B_, and A_3_ Receptors are Involved in Endothelial Protection Induced by Extracellular ATP

To highlight the role adenosine receptors in the endothelial protection by extracellular ATP during hypoxia, pharmacological approach using selective antagonists was performed (Table 1). HUVECs were treated with 10 µM SCH442416 (a selective A_2A_ receptor antagonist), 0.1 µM MRS1754 (selective A_2B_ receptor antagonist), or 10 µM MRS1191 (selective A_3_ receptor antagonist). Then, 10 µM ATP were added prior to two hours of hypoxia. The expression of cleaved caspase 3 was compared to the control (10 µM ATP). The anti-apoptotic effect of 10 µM ATP (cleaved caspase 3 related to control: 20.82 ± 2.43%) was significantly limited in cells after blockade of the A_2A_ receptor (42.80 ± 3.27%), the A_2B_ receptor (39.00 ± 4.25%), and the A_3_ receptor (32.60 ± 3.32%) (*n* = 6, *p* < 0.05) (Figure 3B).

### 2.8. Adenosine Receptor-Mediated Anti-Apoptotic Effect Involves MEK/ERK1/2, PKA, and NOS

**Figure 5:**
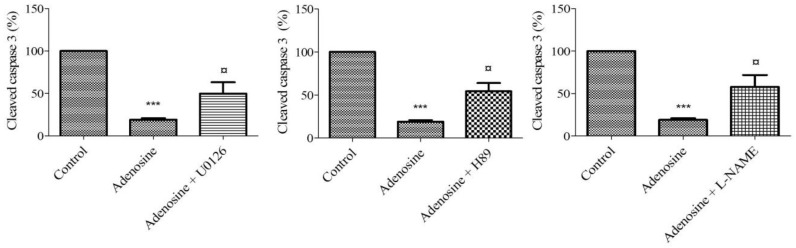
Signaling pathways involved in the anti-apoptotic effect of adenosine. HUVECs were treated with different antagonists: 10 µM U0126 (MEK/ERK1/2), 20 µM H89 (PKA), and 10 µM L-NAME (NOS). Results are expressed as means ± sem (*n* = 6/group) of relative caspase 3 expression (%) in HUVECs after 2 h of hypoxia. ^¤^ *p* < 0.05, compared to 10 µM adenosine group. *** *p* < 0.001 compared to the control.

To highlight the mechanism of adenosine-mediated protection against caspase-3 cleavage, HUVECs were treated with different antagonists (Table 1): 10 µM U0126 (MEK/ERK1/2), 10 µM LY294002 (PI3K), 100 µM 5-HD (mitoK_ATP_ channel), and 10 µM L-NAME (NOS), H89 20 µM (PKA), and 5 µM indomethacin (COX) and then with 10 µM adenosine prior to two hours of hypoxia. The anti-apoptotic effect of adenosine 10 µM was unaffected by blocking COX, PI3K, and mitoK_ATP_ channel pathway (data not shown). The expression of cleaved caspase 3 was performed and compared to the control (adenosine 10 µM). The anti-apoptotic effect of 10 µM adenosine (19 ± 1.82%) was significantly limited in cells by blocking MEK/ERK1/2 (49.75 ± 13.46%), PKA (54.25 ± 9.65%), and the NOS (57.75 ± 14.01%) pathway (*n* = 6, *p* < 0.05) (Figure 5).

## 3. Discussion

In this study, we evaluated the anti-apoptotic effect of extracellular ATP against hypoxic injury in a model of human umbilical vein endothelial cells. The main findings of this study are as follows: (1) Hypoxic stress was associated with a significant release of ATP from endothelial cells. (2) This was associated with an increase in the extracellular concentration of adenosine. (3) The anti-apoptotic effect of extracellular ATP was mediated by both P2 and adenosine receptors. The MEK/ERK1/2, PI3K, mitoK_ATP_ channel, and NOS signaling pathways were involved in the anti-apoptotic effect of extracellular ATP. (4) A_2A_, A_2B_, and A_3_ receptors were involved in the anti-apoptotic effect of ATP. Adenosine-mediated protection involved the MEK/ERK1/2, PKA, and NOS pathways. ATP and adenosine share common signaling pathways such as MEK/ERK1/2 and NOS. 

In our model, endothelial cells were subjected to hypoxic stress (pO_2_ < 2%) [22] for two hours [23]. Compared to normoxic conditions in human umbilical vein blood, hypoxia was considered in HUVEC when an O_2_ content was less than 2% [22]. An important release of ATP rapidly occurred, within the first 15 min after the onset of hypoxia. The concentration of ATP then rapidly decreased after 30 min and was undetectable after 60 min of hypoxia. In response to a large variety of stimuli, ATP can be released from cells [19,24,25,26]. Gerasimovskaya et al. [24] reported that hypoxia induced the release of ATP into the extracellular medium from both adventitial fibroblasts and endothelial cells. They observed an ATP concentration, which peaked at 5 nM after 30 min of hypoxic stress. Urban et al. [19] reported a 2.5-fold increase of extracellular ATP after 10 min of simulated hypoxia. As in our model, they reported a rapid decrease in extracellular ATP after 30 min, which was undetectable after 60 min of hypoxia. Extracellular peak concentration of ATP reached around 108 nM in our model. In plasma sampling, different studies reported concentrations of ATP ranging from 28 to 11,000 nM [27].

With a half-life of approximately 20 s [28], ATP generates ADP, AMP, and adenosine under the action of ectonucleotidases [9,29]. Our result also showed a seven-fold increase in extracellular adenosine in the first stages of hypoxia and a sustained increase at re-oxygenation. Initial studies indicated that the half-life of extracellular adenosine increased fivefold after exposure of the endothelial cells to hypoxic conditions [30]. Conde et al. [31] studied adenosine release during hypoxia from the rat carotid body. Using a nucleotidase inhibitor, they suggested in their model that approximately 40% of extracellular adenosine came from the extracellular catabolism of ATP [31]. Casanello et al. reported adenosine concentrations of 1800 nM after 24 h of hypoxia of endothelial cells [22]. Harrison et al. and Djerada et al. also reported an increase in adenosine concentration (ranging 0.3–18 µM) in interstitial fluid during ischemia/reperfusion in a Langendorff heart model [15,32]. In addition, during hypoxia, an increase in extracellular adenosine production has been reported as a result of increased enzymatic activity of both CD39 and CD73 [30]. Uptake of extracellular adenosine is impaired during hypoxia due to reduced expression of equilibrative nucleoside transporters (ENTs) [22,33,34,35,36]. Lastly, during acute hypoxia, a reduction of adenosine deaminase catabolism was reported [37]. Together, these studies can explain the sustained extracellular adenosine concentrations (Figure 1B, Supplementary file 1).

This current study confirmed the expression of ectonucleotidases, P2, and adenosine receptors in endothelial cells. According to the literature, P2Y_1_, P2Y_2_, P2Y_4_, P2Y_6_, P2Y_11_, P2X_7_, P2X_4_, A_2A_, A_2B_, and A_3_ receptors are expressed in endothelial cells. Wang et al. reported that, among the P2 receptors, the expression of P2X_4_, P2Y_11_, P2Y_1_, and P2Y_2_ was preponderant in endothelial cells [38] whereas, regarding adenosine receptors, A_2A_ and A_2B_ receptors are mostly expressed [9,39]. We also demonstrated the expression of ectonucleotidases CD39 and CD73 in endothelial cells, according to the literature [40].

To demonstrate that ATP and adenosine exert a complementary role in protection against hypoxic stress, we used a non-selective adenosine antagonist (CGS15943) and two P2 receptor antagonists (suramin and PPADS). The ATP concentration used was 10 µM since, at this concentration, expression of cleaved caspase 3 was significantly reduced (Figure 4). First, suramin or PPADS, two P2 antagonists, reduced the protective effect of ATP against hypoxic stress. In agreement with the literature, our results suggest the involvement of P2 in protection against hypoxia [8,12,13,19,20]. The fact that CGS15943 counteracted the anti-apoptotic effect of ATP in response to hypoxia further suggests that both adenosine and P2 receptors play a complementary role in the protective effect against hypoxia [13]. 

Compared to the control, CGS15943 did not significantly increase cleaved caspase 3, while a significant effect was observed with suramin. These results can be explained by the suppression of the protective effect of the endogenous nucleotides released during hypoxia.

The involvement of multiple signaling pathways has been described in the P2Y-mediated cardioprotection against ischemia [8]. The set-up of our protocol using 1 µM CGS15943 and then 10 µM ATP 5 min before hypoxia was aimed at focusing on the protective P2-mediated effect of ATP. In agreement with the literature, the inhibition of MEK/ERK1/2 (U0126), PI3K (LY94002), mitoK_ATP_ channel (5-HD), and NOS (L-NAME) reduced the anti-apoptotic effect of ATP. This emphasizes the major role of these signaling pathways in the protection against hypoxic or ischemic damage [19,41,42,43,44].

Our results also confirm the involvement of the Reperfusion Injury Salvage Kinase (RISK) pathway (both MEK/ERK1/2 and PI3K pathways) in P2-mediated protection. These data are consistent with the well-established role of the RISK pathway in the cardioprotective effects of ischemic preconditioning and post-conditioning [8,40,42,45,46]. 

The inhibition of the mitoK_ATP_ channel by 5-HD reduced the P2-mediated protection of ATP. We suggest that the mitoK_ATP_ channel was involved in the protective effect of the ATP, as previously reported [20]. 

Using L-NAME, which is a NOS inhibitor, we observed a decrease in the protective effect of extracellular ATP. As suggested by Park et al., ATP can activate the P2Y receptors on the endothelium and the downstream Nitric oxid/guanosine monophosphate pathway [44].

On the one hand, among the receptors expressed in our model, only P2Y receptors could be blocked by 10 µM of suramin [47]. The suppression of the ATP-induced protective effect by PPADS, known as a P2Y and P2X receptor antagonist (P2X1,2,3,4,4, and 7 with higher concentrations, i.e., 100 µM) [48], may suggest that P2X receptors are involved especially those expressed in our model such as P2X4 and P2X7 receptors. On the other hand, it has been reported that alpha-methyl ATP, used at concentrations between 0.1 and 300 µM, activates all P2X receptors [49]. Using ascending concentrations of alpha-methyl ATP (10, 100, 1000 µM), Urban did not observe any anti-apoptotic effects in HUVEC cells. In addition, the fact that the anti-apoptotic effect of ATP involved the PI3K, MEK/ERK1/2, mitoKATP, and NOS pathways suggests an engagement of G protein-coupled receptors such as P2Y receptors [19]. Lastly, the P2Y receptors seem to be more likely involved than P2X receptors in the anti-apoptotic effect induced by extracellular ATP. 

Selective antagonist of A_2A_, A_2B_, and A_3_ receptors limited the anti-apoptotic of ATP against hypoxia. A_2A_ receptor has been identified as having cardioprotective and renal protective effect [50,51,52]. A_2B_ receptor has been documented to contribute to cardioprotection [18,50,53]. Other authors also reported A_3_ adenosine receptor-mediated protection of the ischemic heart [18,50,54,55]. 

Adenosine uptake inhibition by ticagrelor and cangrelor is associated with a protective effect in an *in-vivo* model of heart ischemia [56]. Moreover, it has been reported that cardioprotection by cangrelor involved PI3K/Akt and MEK/ERK1/2 pathways [57]. They also reported an involvement of mitoK_ATP_ channel and A_2B_ receptor.

We observed a limited protective effect of adenosine with L-NAME, which is a NOS inhibitor. Our results suggest that the activation of adenosine receptors involved NO production, which is in agreement with Nanhwan et al. [56]. The release of mediators such as nitric oxide might represent a paracrine communication between cardiac endothelial cells and cardiomyocytes, which provides remote protection for cardiac cells. In addition, NO has been found to mediate cardioprotective effects [7,46].

Our results did not highlight the involvement of the COX pathway. This is in contrast with a previous study reporting a cardioprotective effect of ticagrelor dependent on adenosine and the cox pathway [56]. 

Our results demonstrated an involvement of the MEK/ERK1/2, NOS, and PKA pathways in adenosine-mediated protection. It has been reported that adenosine-mediated cardioprotective effects involve different signaling pathways, such as MEK/ERK1/2, PI3K/Akt, NOS, PKA, and COX [50,58]. These discrepancies can be explained by differences between *in-vivo* and/or *in-vitro* models of isolated heart and *in-vitro* cultured HUVECs and differences in the receptor expression. 

## 4. Materials and Methods

### 4.1. Cell Culture

HUVECs were purchased from PromoCell (Sickingenstraße, Heidelberg, Germany) and cultured in endothelial cell growth medium (PromoCell) containing 2% (*v*/*v*) Fetal Coat Serum (FCS), 0.4% (*v*/*v*) endothelial growth supplement: 0.1 ng/mL human Epidermal Growth Factor (EGF), 1.0 μg/mL hydrocortisone, 1 ng/mL human basic fibroblast growth factor (bFGF), 90 µg/mL heparin, and 1 % (*v*/*v*) penicillin/streptomycin (DUTSCHER SAS, Brumath, France) in a fully humidified atmosphere at 37 °C and 5% CO_2_. Confluent cells were detached with the PromoCell detach kit (Sickingenstraße, Heidelberg, Germany) containing 30 mM Hepes, Trypsin/EDTA Solution (0.04%/0.03%), and Trypsin Neutralizing Solution. FCS was reduced to 1% 24 h before the experiment. All experiments were performed on subconfluent endothelial monolayer cells (80%) after the third passage.

### 4.2. Experimental Protocols

Cells were placed into a custom-made hypoxic chamber (Bactron, Sheldon Manufacturing Inc, Cornelius, NC, USA) and exposed to 95% (*v*/*v*) N_2_ and 5% (*v*/*v*) CO_2_ for 2 h at 37 °C. PO_2_ in the hypoxic chamber was reduced to less than 1.5% during the experiments. Control cells were cultured in parallel under normoxic conditions. During the experiments, cells and medium were harvested at different times for cytotoxic analysis (Lactate dehydrogenase LDH activity), Real-time polymerase chain reaction (RT-PCR) analysis, and quantification of nucleotides in the medium. After 2 h of hypoxia, the cleavage of caspase 3 was evaluated by immunoblotting.

Adenosine, AMP, ADP, and ATP were purchased from Sigma-Aldrich (St. Louis, MO, USA). Ligands were added just before hypoxia. P2-receptor antagonists suramin (Sigma-Aldrich, Saint-Louis, MO, USA) and PPADS (Tocris, Bristol, United Kingdom), Adenosine receptor antagonists CGS15943 (Sigma-Aldrich, Saint-Louis, MO, USA), SCH442416 (Sigma-Aldrich, Saint-Louis, MO, USA), MRS1754 (Sigma-Aldrich, Saint-Louis, MO, USA), and MRS1191 (Sigma-Aldrich, Saint-Louis, MO, USA) were added 15 min before hypoxia. The MEK/ERK1/2-inhibitor U0126 (Sigma-Aldrich, Saint-Louis, MO, USA), the PI3K-inhibitor LY294002 (Sigma-Aldrich, Saint-Louis, MO, USA), the PKA-inhibitor H-89 (Sigma-Aldrich, Saint-Louis, MO, USA), the mitoK_ATP_ channel inhibitor (5-HD, Sigma-Aldrich, Saint-Louis, MO, USA), the COX-inhibitor indomethacin (Sigma-Aldrich, Saint-Louis, MO, USA), the nitric oxide synthase (NOS), and the inhibitor L-(NAME Sigma-Aldrich, Saint-Louis, MO, USA) were added 15 min before hypoxia. All these compounds were dissolved in phosphate buffered saline (PBS) or dimethylsulfoxyde (DMSO, Bio Basic Inc, Markham, ON, Canada), according to their solubility. Final DMSO concentration was less than 0.1% in cell culture medium. Working concentrations of inhibitors and antagonists were the same as previously described (Table 1) [12,16,17,19,20].

### 4.3. Quantification of Nucleotides in Extracellular Medium

Culture medium (50 µL) was collected at different times during the course of each experiment. As described, to inhibit ectonucleotidase activity, 75 µL (60% *V*/*V*) of methanol were added and the extracts were frozen at −80° [59,60,61]. Internal standard solution (nicotinamide D4 10 µg L^−1^) was added. Mixtures were evaporated under nitrogen at 40 °C and then reconstituted with 100 μL of ice-cold water (Liquid Chromatography Mass Spectrometry (LC-MS) hypergrade). Liquid chromatography (Hypercarb column 5 µM, 2.1 × 150 mm, ThermoFisher Scientific, San José, CA, USA) coupled with high-resolution mass spectrometer (LC-HRMS) was used for the quantification (ThermoFisher Scientific, San José, CA, USA). High-resolution mass for ATP (C_10_H_16_N_5_O_13_P_3_, negative mode ionization, *m*/*z* 505.98847), for adenosine (C_10_H_13_N_5_O_4_, positive mode ionization, *m*/*z* 268.10403), and for nicotinamide D4 (C_6_H_2_D_4_N_2_O, positive mode ionisation, m/z 127.0804) were used for quantification. TraceFinder Forensic 3.3 was used for LC-MS, library management, acquisition, and processing. These assays were performed as previously described [15,62,63].

### 4.4. RT-PCR

During the course of each experiment, cells were collected at T0 (control), T30, T60, T120, and T240 min. Total RNA was extracted using RNeasy Mini-KitTM (Qiagen, Courtaboeuf, France). cDNA synthesis was obtained from 1 µg RNA using the iScript cDNA synthesis kit (Biorad, Marnes-la-Coquette, France). Real time PCR was performed with Sybr Green PCR reagents and analyzed on an ABI Prism 7500 Fast Real- Time PCR System (Applied Biosystems, ThermoFisher Scientific, San José, CA, USA). PCR was carried out in duplicate for each sample. EEF2 was used as the Housekeeping gene. Results are presented as normalized mRNA levels using the following formula: 2^−ΔΔC^_T_ according to Livak et al. [64]. mRNA expression of ectonucleotidases, adenosine, and P2 receptors was assayed: forward and reverse oligonucleotide primers are described in Supplementary file 4.

### 4.5. Immunoblotting

Samples for Western Blot were collected as follows. After treatment, the cell culture medium was collected and cells were washed in fresh PBS 1X. Protein extraction was performed by adding 100 µL of RIPA Buffer (Sigma-Aldrich) supplemented with protease and phosphatase inhibitor cocktails. Samples were sonicated three times for 10 s and then centrifuged 30 min at 16,000 g at 4 °C. The Bradford assay was performed to quantify proteins in samples. Equal lysed cellular protein (30 µg) were boiled in the sample loading buffer for 5 min before loading on 12.5% sodium dodecyl sulfate–polyacrylamide gel electrophoresis (SDS–PAGE) for electrophoretic separation and subsequently transferred into polyvinylidene difluoride membranes (Immun-Blot PVDF Membrane Bio-Rad, Hercules, CA, USA). After blocking with skimmed milk 5% (*m*/*v*) in TBS-T (Tris-Buffered Saline and TWEEN 20 0.05% (*v*/*v*)) membranes were incubated with the primary antibody overnight at 4 °C. After washing with TBS-T, membranes were incubated with appropriate horseradish peroxidase-labelled secondary antibodies (1 h, 30 min at room temperature) (Supplementary file 5). Immunoreactivity was detected with the Chemiluminescent HRP detection reagent (Millipore, Burlington, VT, USA). Quantification was performed by densitometric analysis using the Quantity One software of ChemiDoc XRS and ImageLab for reprocessing image and quantification (BioRad, Marnes-la-Coquette, France) [15]. 

### 4.6. LDH Activity

The activity of LDH was analyzed using the Pierce LDH Cytotoxicity Assay Kit, according to the manufacturer’s instructions (ThermoFisher, Waltham, MA, USA). Medium was transferred into a new plate and mixed with the Reaction Mixture. After incubation at room temperature for 30 min, the reaction was stopped by adding the Stop Solution. To determine LDH activity, the absorbance were measured at 490 nm and 680 nm by the plate-reading spectrophotometer Victor X3 (Perkin Elmer, Waltham, MA, USA).

### 4.7. Statistical Analysis

Statistical analyses were performed using the Prism 4.00 GraphPad Software, San Diego, CA, USA and R 3.1.4 (The R Foundation for Statistical Computing, http://www.r-project.org). All data are expressed as mean ± sem. Before applying the parametric unpaired t test, the Gaussian distribution of data was assessed by the Shapiro-Wilk normality test and the Kolmogorov-Smirnov test. For non-parametric distribution, a Mann-Whitney test was used. For all significant differences concerning primary endpoints, *a posteriori* powers higher than 80% were checked. Boneferroni correction was applied in the case of multiple comparisons.

All *p* values were two-tailed with statistical significance indicated by a value of *p* < 0.05 [65,66]. 

## 5. Conclusions

The present study shows the complementary role of adenosine and P2 receptors in the endothelial protection induced by extracellular ATP against hypoxic stress. ATP can act directly after its binding to the P2 receptors, which leads to the activation of MEK/ERK1/2, PI3K/Akt, NOS, and the mitoK_ATP_ channel. Adenosine, which is a metabolite of ATP, by binding to A_2A_, A_2B_, and A_3_ receptors, leads to the activation of MEK/ERK1/2, PKA, and NOS. We have identified a complementary role of ATP and adenosine in the anti-apoptotic effect against endothelial cell hypoxia. P2 and adenosine receptors may be novel therapeutic targets to prevent the development of ischemic injury in various organs including the heart, brain, and kidney.

## Figures and Tables

**Table 1:** Panel of inhibitors and antagonists.

Compounds Name	Target	Concentration	Reference
Suramin	P2 receptors antagonist	10 µM	Wee et al. [12]
PPADS	P2 receptors antagonist	10 µM	Wee et al. [12]
CGS 15943	Adenosine receptors antagonist	1 µM	Avanzato et al. [16]
SCH442416	selective receptor antagonist A2A	10 µM	Yu et al. [17]
MRS1754	selective receptor antagonist A2B	0.1 µM	Salie et al. [18]
MRS1191	selective receptor antagonist A3	10 µM	Salie et al. [18]
U0126	ERK1/2 inhibitor	10 µM	Urban et al. [19]
LY294002	PI3K inhibitor	10 µM	Urban et al. [19]
5-HD	mitoK^+^ATP inhibitor	100 µM	Millart et al. [20]
L-NAME	NOS inhibitor	10 µM	Millart et al. [20]
H89	PKA inhibitor	20 µM	Millart et al. [20]
indomethacin	COX inhibitor	5 µM	Alm et al. [21]

This table shows the different inhibitors and antagonists used for experiments. Concentrations and references are quoted.

## Supplementary Materials

Supplementary materials can be found at https://www.mdpi.com/1422-0067/20/6/1446/s1. Supplementary file 1: Extracellular kinetics of nucleotides and adenosine during hypoxia and re-oxygenation. Data are expressed relative to baseline as log_10_ (means ± sem) (n = 6/group). Supplementary file 2: LDH activity of endothelial cells after 2 h of hypoxia. Data are means ± sem (*n* = 6/group). * *p* < 0.05, compared to the negative control group. Supplementary file 3: Inhibitors of signaling pathways. HUVECs were treated with different antagonists: 10 µM U0126 (MEK/ERK1/2), 10 µM LY294002 (PI3K), 20 µM H89 (PKA), 10 µM L-NAME (NOS), and 100 µM 5-HD (mitoKATP). Results are expressed as means ± sem (*n* = 6/group) of relative caspase 3 expression (%) in HUVECs after 2 h of hypoxia. Supplementary file 4: Forward and reverse oligonucleotide primers for RT-PCR analysis. Supplementary file 5: Primary and secondary antibodies for immunoblotting analysis.
