# Supplementary material for: Complementary Role of P2 and Adenosine Receptors in ATP Induced-Anti-Apoptotic Effects Against Hypoxic Injury of HUVECs
Source: Int J Mol Sci. 2019 Mar 22;20(6):1446. doi: 10.3390/ijms20061446 (PMC6470483; doi:10.3390/ijms20061446)
Supplement: Supplementary file 1 [file ijms-20-01446-s001.zip › SF4.pdf]

Supplemental file 4: Forward and reverse oligonucleotide primers for RT-PCR analysis.

Oligo name	Oligo name	Sequence
EEF2	sense primer	GGC-CCT-CTT-ATG-ATG-TAT-ATT-TCC
	antisense primer	CTG-ACC-TTC-AGG-CCA-GT
NT5E	sense primer	GGA-TAC-ACT-TCC-AAA-GAA-ACC-C
	antisense primer	ATC-TAC-TTC-AGG-TTG-TAA-TGC-AG
ENTPD1	sense primer	CTA-TCA-ACT-ATC-TGC-TGG-GCA-A
	antisense primer	GCT-CCA-AAG-GTT-TCC-TGA-TTA-T
P2Y1	sense primer	CGT-GCT-GGT-GTG-GCT-CAT-T
	antisense primer	GGA-CCC-CGG-TAC-CTG-AGT-AGA
P2Y2	sense primer	CGA-GGA-CTT-CAA-GTA-CGT-GCT-G
	antisense primer	GTG-GAC-GCA-TTC-CAG-GTC-TTG-A
P2Y4	sense primer	TGT-CCT-TTT-CCT-CAC-CTG-CAT
	antisense primer	TGC-CCG-AAG-TGG-GTG-G
P2Y6	sense primer	CCT-GCC-CAC-AGC-CAT-CTT
	antisense primer	GGC-TGA-GGT-CAT-AGC-AGA-CAG-TG
P2Y11	sense primer	GTT-GGT-GGC-CAG-TGG-TGT-G
	antisense primer	TTG-AGC-ACC-CGC-ATG-ATG-T
P2X1	sense primer	TCT-CTC-CCC-AGG-CTT-CAA-CTT
	antisense primer	GAG-GTG-ACG-GTA-GTT-GGT-CCC
P2X4	sense primer	CAT-CAT-CCC-CAC-TAT-GAT-CAA-CA
	antisense primer	AGC-ACG-GTC-GCC-ATG-C
P2X7	sense primer	ATC-GGC-TCA-ACC-CTC-TCC-TAC
	antisense primer	CTG-GAG-TAA-GTG-TCG-ATG-AGG-AAG
CASPASE3	sense primer	TGT-GAG-GCG-GTT-GTG-GAA-GAG-T
	antisense primer	AAT-GGG-GGA-AGA-GGC-AGG-TGC-A
