# Supplementary material for: Complementary Role of P2 and Adenosine Receptors in ATP Induced-Anti-Apoptotic Effects Against Hypoxic Injury of HUVECs
Source: Int J Mol Sci. 2019 Mar 22;20(6):1446. doi: 10.3390/ijms20061446 (PMC6470483; doi:10.3390/ijms20061446)
Supplement: Supplementary file 1 [file ijms-20-01446-s001.zip › SF5.pdf]

Supplemental file 5: Primary and secondary antibodies for immunoblotting analysis.

Proteins	Primary antibody	Secondary antibody
Cleaved caspase 3	Anti-Cleaved Caspase-3 (Asp175) (5A1E) Rabbit mAb (Cell signaling #9664) - 1:1000	Goat anti-rabbit, HRP-linked antibody (Cell signaling #7074) – 1:3000
β-Actin	Anti-β-Actin Mouse mAb (Sigma-aldrich #A1978) – 1:4000	Goat anti-mouse, HRP-linked antibody (Abcam #ab97023) – 1:4000
GSK-3β	Anti-GSK-3β (27C10) Rabbit mAb (Cell signaling #9315) – 1:1000	Goat anti-rabbit, HRP-linked antibody (Cell signaling #7074) – 1:3000
Phospho-GSK-3β	Anti-Phospho-GSK-3β (Ser9) (D2Y9Y) Mouse mAb (Cell signaling #14630) – 1 :1000	Goat anti-mouse, HRP-linked antibody (Abcam #ab97023) – 1:4000
