# Supplementary figures and images for: Complementary Role of P2 and Adenosine Receptors in ATP Induced-Anti-Apoptotic Effects Against Hypoxic Injury of HUVECs
Source: Int J Mol Sci. 2019 Mar 22;20(6):1446. doi: 10.3390/ijms20061446 (PMC6470483; doi:10.3390/ijms20061446)

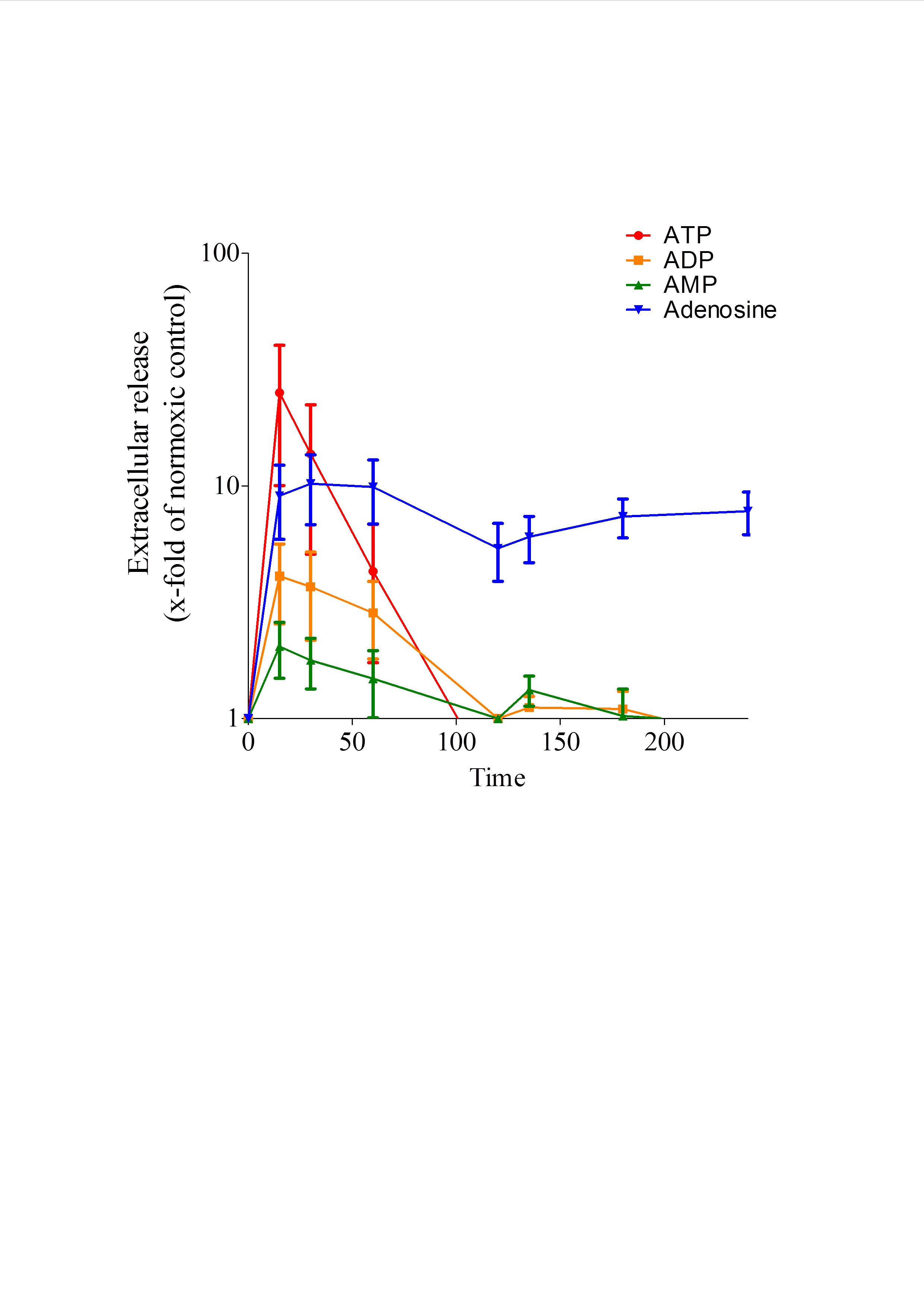

Supplement: Supplementary file 1 [file ijms-20-01446-s001.zip › SF1.jpg]

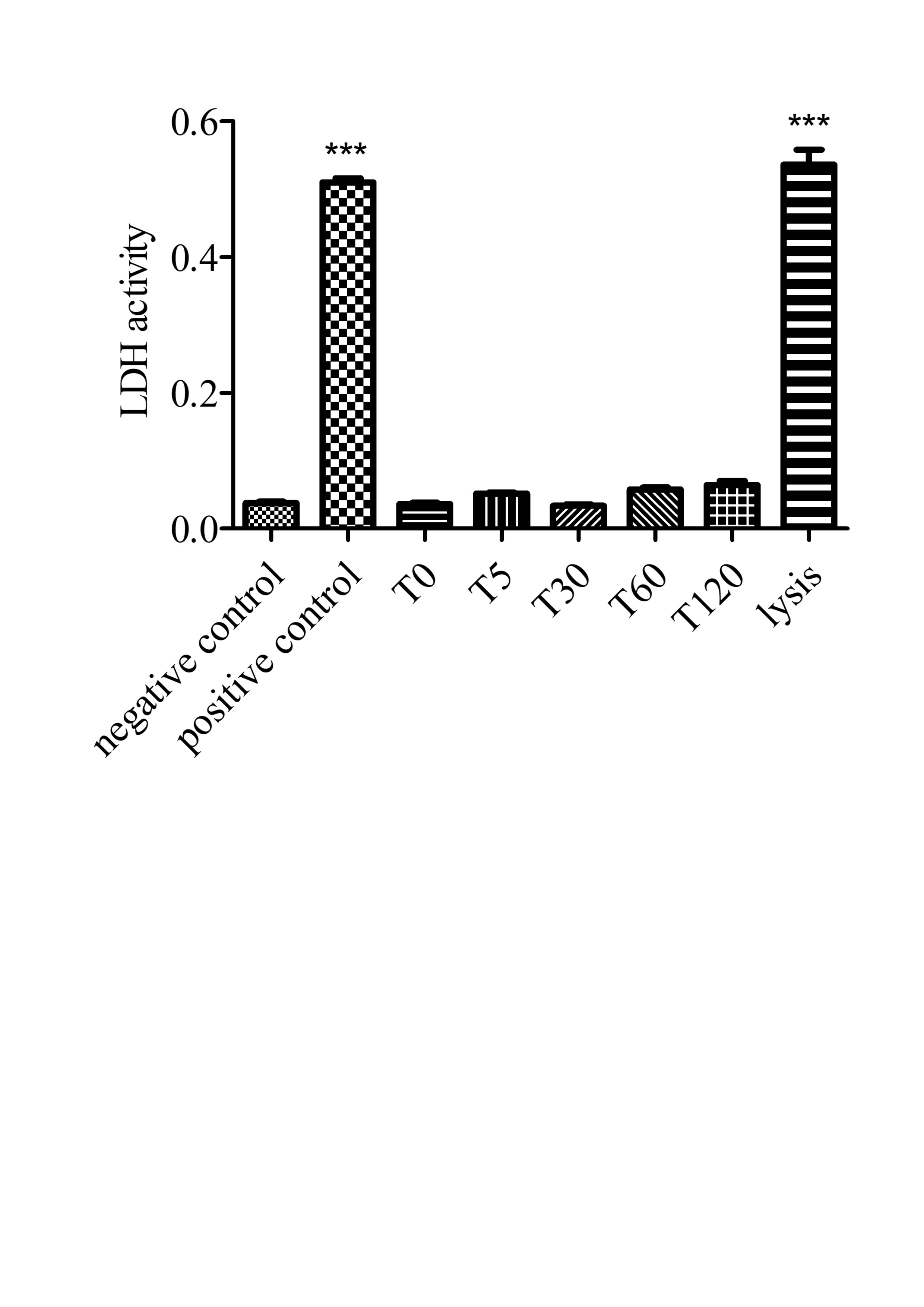

Supplement: Supplementary file 1 [file ijms-20-01446-s001.zip › SF2.jpg]

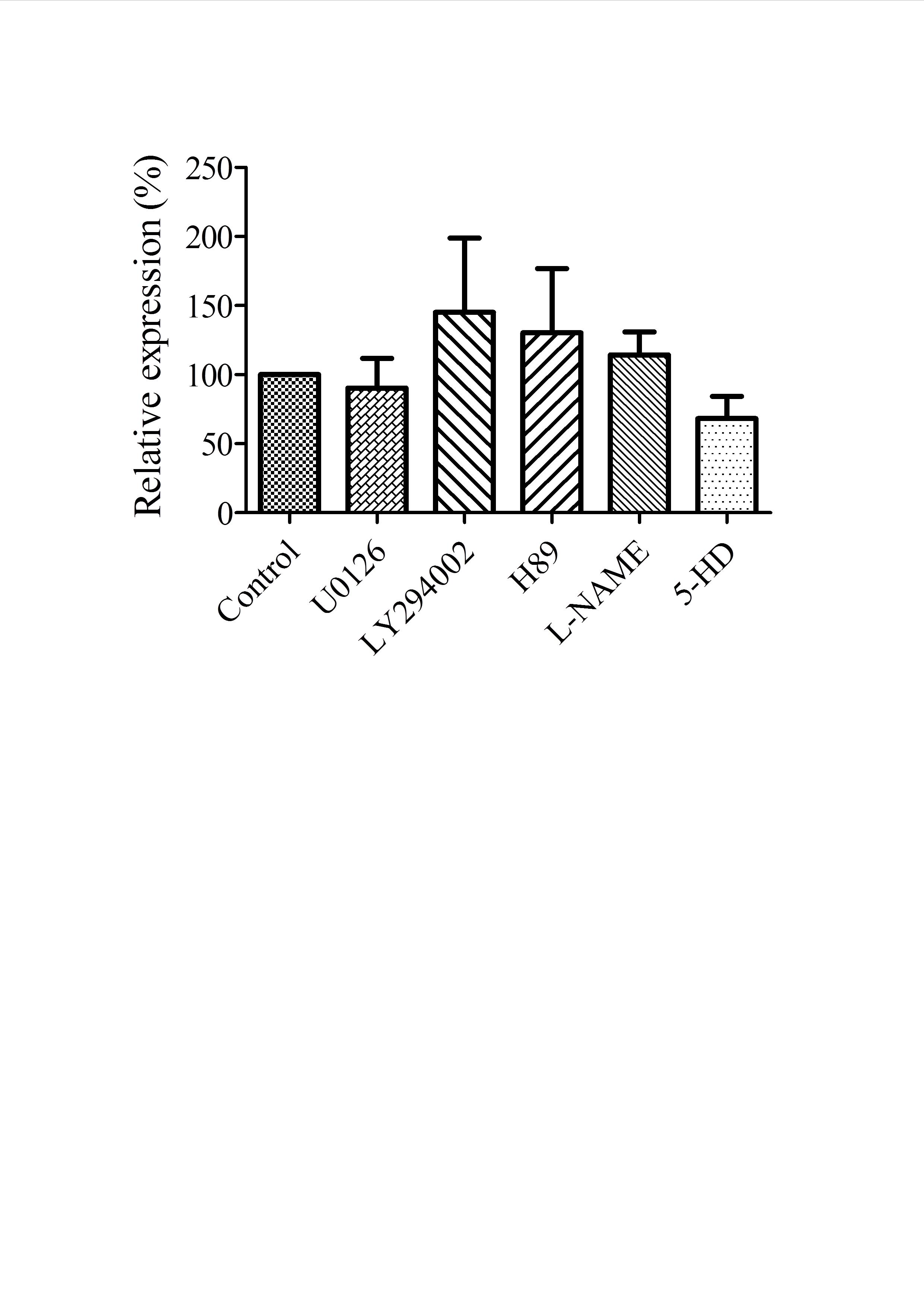

Supplement: Supplementary file 1 [file ijms-20-01446-s001.zip › SF3.jpg]
